# Interoceptive awareness in a Southeastern US college sample: validation of the multidimensional assessment of interoceptive awareness – version 2

**DOI:** 10.1186/s13104-024-06894-6

**Published:** 2024-08-27

**Authors:** Harrison E. Chapman, Alan E. Stewart

**Affiliations:** Department of Counseling and Human Development Services, University of Georgia, Athens, GA USA

**Keywords:** Interoception, Body-awareness, Somatic psychotherapies, Mind-body, Mindfulness

## Abstract

**Objective:**

The Multidimensional Assessment of Interoceptive Awareness, version 2 (MAIA-2) is a commonly utilized self-report instrument to assess individuals’ ability to perceive bodily sensations. The MAIA-2 has displayed variable psychometric properties across samples. Thus, we examine the psychometric properties of the MAIA-2 in a Southeastern United States college sample.

**Participants:**

Our studies consisted of 710 (study 1) and 66 (study 2) college students.

**Methods:**

Study 1 used a cross-sectional research design where we investigated the factor structure, and measurement invariance (e.g., measured similarly across genders). Study 2 examined the test-retest reliability across a three-week period.

**Results:**

The MAIA-2 displayed adequate to good internal consistencies and factor loadings, strict invariance, and questionable temporal stability.

**Conclusion:**

The MAIA-2 demonstrates adequate psychometric properties in this college sample that were similar to the original MAIA sample characteristics. Contextual and cultural factors may influence the subjective experience of interpreting bodily sensations.

**Supplementary Information:**

The online version contains supplementary material available at 10.1186/s13104-024-06894-6.

## Introduction

University counseling centers (UCC) have hired more therapists and emphasized outreach programming to accommodate the increased demand for services and to meet the continued rise of mental health concerns for college students [1–3]. Mindfulness and body-based psychotherapies, which have demonstrated positive results for depression, anxiety, and self-compassion [4–6], provide one such way UCCs meet this need. The effectiveness of these interventions may be assessed through changes in interoceptive awareness [7, 8].

Interoceptive awareness is characterized by the self-reported ability to consciously perceive and judge internal bodily sensations and is associated with various mental health conditions [9–13]. For example, college students’ interoceptive awareness was related to emotional eating [14, 15], body appreciation, intuitive eating [16], and anxiety [17]. The Multidimensional Assessment of Interoceptive Awareness Version 2 (MAIA-2) is a 37-item, eight-dimension measure of self-reported interoceptive awareness [13], is the second most used measure of interoceptive awareness [18], and has demonstrated varying psychometric properties across different samples [12, 13, 18–29]. The MAIA-2’s constructs capture a person’s awareness, trust, and tendency to listen to bodily sensations, a tendency to avoid or be distressed with bodily discomfort, attention regulation toward bodily sensations, awareness of physiological sensations with emotional experiences, and ability to regulate via bodily sensation [12].

Mehling and colleagues’ original sample consisted of largely white female participants, over half completed graduate education, and all engaged in some form of somatic practice (e.g., yoga) [12]. The MAIA-2 sample included participants visiting the Science Museum of London, UK, who were 47% female and 60% native English speakers, but no information about fluency or acculturation for non-native speakers was collected [13]. The MAIA’s extensive use has led to validations in various populations. Researchers from six countries (Portugal [24], Japan [25, 26], Chile [27], Italy [23], Lithuania [22], and Columbia [28]) evaluated the psychometric properties of the MAIA in different college samples. These studies demonstrated factor structures that included the observation of eight [23, 27], seven [24], and six dimensions [22, 25, 26], with questionable to acceptable model fit and internal consistency values (e.g., α ranging from 0.40 [27] to 0.87 [24–26]). A full review of these validations is beyond the scope of this report and for further interest see Todd et al. [29] and the MAIA website [30].

There is a need to validate the MAIA-2 in a US college population, given that (1) college students’ mental health is an increasing concern [1–3], (2) difficulties with interoceptive awareness are associated with various mental health conditions [14–17], (3) college student samples demonstrated different factor structures [23–28], (4) the MAIA-2 is commonly used [18], and (5) to answer the call for replication studies [31]. Therefore, we aim to examine the psychometric properties of the MAIA-2 in a United States college sample by examining the internal validity, factor structure, and test-retest reliability.

## Main text

### Study 1

#### Methods

Our university’s Institutional Review Board (STUDY00006902) reviewed and approved the project measures and procedures. The study included 710 undergraduate and graduate students from the University of Georgia. Participants were majority white (63.2%) and female (69.6%), with a mean age of 23.77 years (SD = 7.29). The participants provided their informed consent and then completed the MAIA-2, demographics, and other measures beyond the scope of this study. Complete demographic information appears in supplementary material Table 1.

#### Data analysis

All analyses were performed with R (version 4.2.2) [32]. Descriptive data, distribution skewness, and kurtosis were obtained, and missing items were assessed using Little’s Missing at Random (MCAR) [33] and a maximum likelihood procedure in the MICE package [34]. The data were evaluated for internal consistency as measured by Cronbach’s alpha [35], and McDonald’s Omega [36], using the Coefficient alpha package [37], where > 0.70 is acceptable, > 0.8 is good, and 0.9 > is excellent [38].

We assessed the fit of the original eight-factor measurement model [12, 13] using confirmatory factor analysis (CFA) with the maximum likelihood estimation with robust standard errors and a Satorra-Bentler scaled test statistic. Goodness-of-fit of the model was evaluated using the Comparative Fit Index (CFI) and Tucker Lewis Index (TLI) (good fit $\:\ge\:$ 0.95), Root Mean Square Error of Approximation (RMSEA; good fit $\:\le\:\:$0.05; adequate fit $\:\le\:$ 0.08), and Standardized Root Mean Square Residual (SRMR; acceptable fit $\:\le\:$ 0.08) [39, 40]. Modification indices were examined to assess for improved model fit. All factor calculations were completed using the Lavaan package in R [41].

Thirteen participants identified as gender-diverse, with two missing gender identities. Due to this, the invariance testing was conducted on binary gender identities (*n* = 695; males [*n* = 494]; females [*n* = 201]) to ensure adequate numbers in each group, while all other analyses included the full sample. We examined multiple nested models in a forward approach, using (1) configural invariance (i.e., assessing if factor structure is equal across groups), (2) metric invariance (i.e., assessing if factor loadings are equal across groups), and (3) scalar invariance (i.e., assessing if item intercepts are equal across groups) [40, 42, 43]. Invariance was assessed using the cutoff criteria of ΔCFI < 0.01 and ΔRMSEA < 0.15 [40, 42, 43].

#### Results

The sample had a total of 17 (0.06%) missing items. Little’s MCAR test suggested that the data were missing at random; thus, the 17 missing items were estimated using the MICE [34] package in R.

##### Internal consistency

The results suggested good values for the internal consistencies of the subscale items for ND, NW, AR, SR, BL, and TR and adequate internal consistency values for the NT and EA subscales (see Table 1). Students endorsed a tendency to use distraction as a coping strategy and trust their bodies, as indicated by the percentile ranges.

**Table 1:** Results of the Confirmatory Factor Analysis and Subscales means, SD, and internal consistency

		Items				Dimensions				
**Dimension** Items		Std. Est.	SE	Z-value		Mean (SD)	10th Percentile	90th Percentile	Cronbach’s Alpha	McDonald’s Omega
**Noticing**						3.43 (0.78)	2.50	4.50	0.72	0.72
1. When I am tense, I notice where the tension is located in my body		0.67	0.05	16.11***						
2. I notice when I am uncomfortable in my body.		0.68	0.04	14.66***						
3. I notice where in my body I am comfortable.		0.59	0.05	13.75***						
4. I notice changes in my breathing, such as whether it slows down or speeds up		0.58	0.04	15.07***						
**Not Distracting**						1.69 (0.71)	0.83	2.50	0.71	0.83
5. I ignore physical tension or discomfort until they become more severe.		0.43	0.05	9.57***						
6. I distract myself from sensations of discomfort.		0.63	0.04	14.82***						
7. When I feel pain or discomfort, I try to power through it.		0.50	0.04	11.07***						
8. I try to ignore pain.		0.58	0.04	13.06***						
9. I push feelings of discomfort away by focusing on something		0.83	0.03	21.56***						
10. When I feel unpleasant body sensations, I occupy myself with something else so I don’t have to feel them.		0.80	0.04	21.22***						
**Not Worrying**						2.51 (0.81)	1.40	3.60	0.81	0.80
11. When I feel physical pain, I become upset.		0.65	0.05	16.28***						
12. I start to worry that something is wrong if I feel any discomfort.		0.67	0.04	17.56***						
13. I can notice an unpleasant body sensation without worrying about it.		0.53	0.04	12.27***						
14. I can stay calm and not worry when I have feelings of discomfort or pain.		0.63	0.04	16.05***						
15. When I am in discomfort or pain, I can’t get it out of my mind.		0.66	0.05	15.95***						
**Attention Regulation**						3.06 (0.75)	2.14	4.00	0.86	0.86
16. I can pay attention to my breath without being distracted by things happening around me.		0.58	0.05	14.24***						
17. I can maintain awareness of my inner bodily sensations even when there is a lot going on around me.		0.64	0.04	16.93***						
18. When I am in conversation with someone, I can pay attention to my posture.		0.57	0.04	14.78***						
19. I can return awareness to my body if I am distracted.		0.73	0.03	20.05***						
20. I can refocus my attention from thinking to sensing my body.		0.73	0.04	19.71***						
21. I can maintain awareness of my whole body even when a part of me is in pain or discomfort		0.62	0.04	15.00***						
22. I am able to consciously focus on my body as a whole.		0.62	0.04	15.71***						
**Emotional Regulation**						3.49 (0.82)	2.40	4.60	0.79	0.79
23. I notice how my body changes when I am angry		0.51	0.05	12.75***						
24. When something is wrong in my life, I can feel it in my body.		0.47	0.05	11.24***						
25. I notice that my body feels different after a peaceful experience.		0.73	0.05	18.16***						
26. I notice that my breathing becomes free and easy when I feel comfortable.		0.71	0.04	17.88***						
27. I notice how my body changes when I feel happy / joyful.		0.80	0.04	23.39***						
**Self-Regulation**						2.97 (0.88)	1.75	4.00	0.82	0.82
28. When I feel overwhelmed, I can find a calm place inside.		0.65	0.05	16.96***						
29. When I bring awareness to my body, I feel a sense of calm.		0.74	0.04	18.22***						
30. I can use my breath to reduce tension.		0.65	0.04	16.13***						
31. When I am caught up in thoughts, I can calm my mind by focusing on my body/breathing.		0.68	0.05	17.14***						
**Body Listening**						2.68 (1.01)	1.33	4.00	0.84	0.84
32. I listen for information from my body about my emotional state.		0.79	0.04	23.38***						
33. When I am upset, I take time to explore how my body feels.		0.79	0.04	23.75***						
34. I listen to my body to inform me about what to do.		0.74	0.04	20.04***						
**Trusting**						3.53 (0.95)	2.33	5.00	0.88	0.90
35. I am at home in my body.		0.86	0.04	24.02***						
36. I feel my body is a safe place.		0.92	0.04	28.30***						
37. I trust my body sensations.		0.67	0.04	16.61***						
**Modification Covariances**										
Items 5 and 6		0.32	0.04	6.31***						
Items 7 and 8		0.48	0.03	7.98***						
Items 13 and 14		0.41	0.05	6.20***						
Items 16 and 17		0.40	0.05	6.58***						
Items 19 and 20		0.38	0.03	5.94***						
Items 21 and 22		0.31	0.05	4.28***						
Items 30 and 31		0.58	0.05	8.19***						

*Notep*-value < 0.05 = *, *p*-value < 0.001 = **, *p*-value < 0.001 = ***

##### Confirmatory factor analysis

The results show acceptable measures of model fit using the RMSEA (0.60 [0.057, 0.063]) and the SRMR (0.067) and questionable measures of fit indices for CFI (0.855), TLI (0.839), and x^2^ = 1812.799, df = 601, *p* < 0.001. Standardized factor loadings indicate a meaningful level of item contribution to each factor (0.51–0.92). Table 2 in the supplementary material includes all factor loadings for the MAIA-2 measurement model.

We examined modification indices (MI) and included seven covariance terms across 14 items to improve the model fit. The results of the MAIA-2 with MI exhibited good measures of fit indices for RMSEA (0.046 [0.043, 0.049]) and SRMR (0.063) and improved measures of fit indices for CFI (0.917), TLI (0.906), and x^2^ = 1304.558, df = 594, *p* < 0.001. The results suggest adequate standardized factor loadings (0.41–0.93). Table 1 displays all factor loadings with MI included.

##### Measurement invariance

Table 2 shows the changes in chi-square, CFI, and RMSEA as model constraints were added. The sample met the recommended ΔCFI < 0.01 and ΔRMSEA < 0.15 and supported equal factor structure (configural), item loadings (metric), and intercepts (scalar) across groups. Thus, the model fit for the MAIA-2 demonstrated scalar invariance between men and women in our sample.

**Table 2:** Measurement invariance of the MAIA-2 for women (*n* = 494) and men (*n* = 201)

Invariance	df	χ^2^	Δχ^2^	Δdf	CFI	RMSEA	ΔCFI	ΔRMSEA
Configural	1188	1894.757	-	-	0.914	0.046	-	-
Metric	1217	1938.381	43.624	29	0.913	0.046	0.001	0.000
Scalar	1246	2026.297	87.916	29	0.907	0.047	0.006	0.000

*Note* Df = Degrees of freedom; χ^2^  = Chi-square; Δχ^2^ = Change in Chi-square; Δdf = change in degrees of freedom; CFI = Comparative Fit Index; RMSEA = Root Mean Square Error of Approximation; ΔCFI = Change in Comparative Fit Index; ΔRMSEA = Change in Root Mean Square Error of Approximation

#### Discussion

The results from Study 1 suggested that the MAIA-2 has generally good psychometric properties, with some qualifications. First, the items appeared internally consistent with how participants understood and responded, which has varied in college sample validation studies [23:29]. Unlike previous studies [12, 13, 29], the NW and ND items demonstrated consistent participant responses. Second, the items displayed adequate coverage of multiple dimensions of interoceptive awareness, with questionable- to good-fit for the measurement model and comparable with the original studies [12, 13]. Lastly, the results demonstrated invariance between men’s and women’s responses, which means that the factor analytic results do not differ according to the student’s gender.

### Study 2

#### Materials

We examined the test-retest reliability of the MAIA-2 using a three-week test-retest interval. The University of Georgia’s IRB approved the research (PROJECT00005184). The material consisted of a printed, hard-copy packet that contained the MAIA-2 and a demographic form. The participants were recruited through a university research pool, partially fulfilled the students’ academic research requirements, and were required to schedule both administration dates. Sixty-six participants completed the research in groups ranging from four to twenty people. At the first session, the participants were provided an overview of the study and written informed consent, which was signed before administering the measures. In the second session, participants completed the same measures and were provided a written debriefing statement summarizing the purposes of the research.

#### Data analysis

We used R (version 4.2.2) to analyze the data [32]. Internal consistency was evaluated using Cronbach’s alpha [35], and McDonald’s Omega [36], using the Coefficient alpha package [37], where > 0.70 is acceptable, > 0.8 is good, and 0.9 > is excellent [38]. Reliability was assessed using Pearson’s correlation between times 1 and 2, where > 0.70 is considered adequate and > 0.80 is considered good. We conducted a paired t-test of the subscales over the three-week interval to further assess stability.

#### Results

The demographic makeup of the test-retest participants was predominantly white (69.23%) and female (86.0%), with a mean age of 21.05 (SD = 1.35). Participants’ scores on the subscales displayed adequate (NT subscale) to good internal consistency. The Pearson correlations of the participants’ scores were statistically significant. Subscales ND (*r* = 0.67) and NW (*r* = 0.66) exhibited questionable test-retest reliability. Only the NW dimension differed in a statistically meaningful way from time 1 (M = 2.12, SD = 0.78) to 2 (M = 2.30, SD = 0.76); t (65) = 2.25, *p* = 0.03. All other subscales demonstrate adequate temporal stability. See Table 3 for all reported statistics.

**Table 3:** Time 1 and Time 2: internal consistency, test-retest Pearson Correlations, and T-tests

Scale	Time 1 Alpha	Time1 Omega	Time 2 Alpha	Time 2 Omega	Test-retest correlation	Explained Variance	Time 1 Mean(sd)	Time 2 Mean(sd)	T-Test (65)	*P*-value
NT	0.73	0.74	0.75	0.75	0.70***	49.0%	3.48(0.72)	3.35(0.67)	2.00	0.05
ND	0.89	0.89	0.91	0.91	0.67***	44.9%	1.94(0.85)	1.95(0.92)	0.11	0.91
NW	0.81	0.82	0.74	0.74	0.66***	43.6%	2.12(0.78)	2.30(0.76)	2.25	0.03*
AR	0.82	0.82	0.89	0.89	0.73***	54.3%	2.86(0.67)	2.80(0.83)	0.90	0.37
EA	0.84	0.83	0.91	0.91	0.79***	62.4%	3.54(0.83)	3.45(0.92)	1.33	0.19
SR	0.87	0.88	0.88	0.89	0.73***	63.3%	2.81(0.96)	2.76(1.01)	0.64	0.53
BL	0.89	0.90	0.90	0.90	0.75***	56.3%	2.51(1.09)	2.49(1.12)	0.16	0.88
TR	0.83	0.86	0.93	0.93	0.73***	53.3%	3.53(0.99)	3.54(1.01)	0.06	0.96

*Notep*-value < 0.05 = *, *p*-value < 0.01 = **, *p*-value = < 0.001

NT = Noticing; NA = Not Distracting; NW = Not Worrying; AR = Attention Regulation; EA = Emotional Awareness; SR = Self-Regulation; BL = Body Listening; TR = Trusting

#### Discussion

The results of study 2 indicated that respondents’ scores on the dimensions of the MAIA-2 had acceptable internal consistency characteristics and variable temporal stability. Specifically, the NW subscale data suggested that respondents consistently interpret and respond to items for this scale but report variation in their location (i.e., mean values). Moreover, the NW mean value showed a tendency to experience emotional distress by physical discomfort.

## General discussion

Our sample demonstrated (1) interpretability of the items, (2) good model fit with modifications and strict invariance between men and women, and (3) generally acceptable temporal stability. The two studies are informative because screening and assessment for attention, concentration, and related mind-body processing phenomena frequently occur during peoples’ college years.

The CFA results show a good degree of fit and retention of all items on their original factors in this college sample, with some minor modifications in the items’ covariances and similar properties to Mehlings et al.’s results [12, 13]. Previous validation studies with college samples demonstrate different factor structures and item retention, such as six factors with Japanese [25, 26] and Lithuanian [22] samples, while a Portuguese sample [24] resulted in seven dimensions.

Our sample appeared to share seemingly similar sample sociodemographics of the MAIA [12], and MAIA-2 [13] (i.e., predominately English-speaking white female participants) and confirmed the factor structure of the measure, providing additional consideration that cultural and contextual factors may impact the subjective experience of interoceptive awareness. Cultural differences have been theorized as influential factors in the subjective nature of interpreting bodily signals [25, 26], thus potentially impacting the psychometrics of the MAIA-2.

Regarding reliability, the findings support consistent interpretation and responses to all dimensions of the MAIA-2. The results implied adequate temporal stability except for the NW and ND subscales, displaying similar results as a Dutch non-clinical sample [44]. The college respondents reported fluctuating tendencies to use distraction to cope with emotional distress from physical discomfort. The NW dimension has displayed negative associations with pain catastrophizing [11, 12], state and trait anxiety [11, 12, 45], somatosensory amplification, and neuroticism [46]. The ND dimension was negatively correlated with difficulty identifying feelings [46], anxiety [11, 12, 17], and positively associated with mindfulness [11, 12]. This might imply that the NW and ND scales are more analogous to state characteristics that contextual factors (e.g., time of the semester) influence and would help explain the variability in its performance across time. Students’ tendency to worry and utilize distraction to regulate is consistent with increased rates of anxiety and other mental health concerns on college campuses [1, 2]. This sample also strongly endorsed a tendency to notice, attend to, assign emotions from, and trust their bodily signals. These findings warrant further investigations into the utilization of somatic therapies, such as somatic reappraisal and mindfulness via interoceptive attention [47].

## Limitations

First, we did not evaluate the convergent or discriminate validity. Second, the data was limited to samples from a single US college, raising questions of external validity. Third, the study did not measure participants’ experiences with mind-body practices or mental health conditions; therefore, we could not assess if these practices or conditions influenced the college students’ scores. Beyond these limitations, the MAIA-2 appears to be a generally good measure of interoceptive awareness and one to which people can consistently respond over time.

### Electronic supplementary material

Below is the link to the electronic supplementary material.

Supplementary Material 1

## Data Availability

Contact the corresponding author with questions or requests regarding the data in this project.

## Abbreviations

NT: Noticing
ND: Not-distracting
NW: Not-worrying
AR: Attention regulation
EA: Emotional awareness
SR: Self-regulation
BL: Body listening
TR: Trusting
