# Supplementary material for: Interoceptive awareness in a Southeastern US college sample: validation of the multidimensional assessment of interoceptive awareness – version 2
Source: BMC Res Notes. 2024 Aug 27;17:236. doi: 10.1186/s13104-024-06894-6 (PMC11348765; doi:10.1186/s13104-024-06894-6)
Supplement: Supplementary file 1 — Supplementary Material 1 [file 13104_2024_6894_MOESM1_ESM.docx]

| **Supplementary Table 1** Participants reported Age, Gender identity, and Race for Study 1 and Study 2 | | | |
| --- | --- | --- | --- |
| Demographics |  | Study Samples | |
|  |  | Study 1  (n=710) | Study 2  (n=66) |
| Mean Age in years (SD) |  | 23.77(7.29) | 21.05(1.35) |
|  |  |  |  |
| Gender Identify (%) |  |  |  |
| Male |  | 201 (28.3%) | 9 (13.64%) |
| Female |  | 494 (69.6%) | 56 (86.0%) |
| Transman |  | 1 (0.14%) | 1 (0.3%) |
| Transwoman |  | 0 (0%) | 0(0%) |
| Genderqueer/Gender non-conforming |  | 10 (1.4%) | 1(1.52%) |
| Different Identify |  | 0 (0%) | 0 (0%) |
| Questioning |  | 1 (0.14%) | 0 (0%) |
| Other |  | 1 (0.14%) | 0 (0%) |
|  |  |  |  |
| Race(%) |  |  |  |
| African American/Black |  | 37(5.2%) | 9 (13.85%) |
| Arab American |  | 4(0.6%) | 1(0.3%) |
| Asian American |  | 66(9.3%) | 7 (10.77%) |
| Caribbean Islander |  | 2(0.28%) | 0 (0%) |
| White |  | 449(63.2%) | 45 (69.23%) |
| Continental India |  | 8(1.1%) | 2(0%) |
| Latinx |  | 33(4.6%) | 2 (3.08%) |
| Native American |  | 0(0%) | 0(0%) |
| Pacific Islander |  | 0(0%) | 0(0%) |
| Multiracial |  | 54(7.6%) | 2 (3.04%) |
| Other |  | 48(6.8%) | 0 (0%) |
| *Note. All options for participants are provided in the table. Participants were offered to select more than one race and/or gender identity. For study 1, there were incomplete fields for age (n=34), gender identity (n=2), and race (n=8). Thus, percentages may not equal 100.* | | | |

| **Supplementary Table 2** Results of the Confirmatory Factor Analysis without Modification Indices | | | | |  |
| --- | --- | --- | --- | --- | --- |
|  |  | Items | | |  |
| **Dimension**  Items |  | Std. Est. | SE | Z-value |  |
| **Noticing** |  |  |  |  |  |
| 1. When I am tense, I notice where the tension is located in my body |  | 0.67 | 0.05 | 16.24*** |  |
| 2. I notice when I am uncomfortable in my body. |  | 0.68 | 0.04 | 14.49*** |  |
| 3. I notice where in my body I am comfortable. |  | 0.59 | 0.05 | 13.76*** |  |
| 4. I notice changes in my breathing, such as whether it slows down or speeds up |  | 0.58 | 0.04 | 15.04*** |  |
| **Not Distracting** |  |  |  |  |  |
| 5. I ignore physical tension or discomfort until they become more severe. |  | 0.52 | 0.05 | 12.63*** |  |
| 6. I distract myself from sensations of discomfort. |  | 0.66 | 0.04 | 16.49*** |  |
| 7. When I feel pain or discomfort, I try to power through it. |  | 0.61 | 0.04 | 14.51*** |  |
| 8. I try to ignore pain. |  | 0.67 | 0.04 | 16.54*** |  |
| 9. I push feelings of discomfort away by focusing on something |  | 0.77 | 0.03 | 20.48*** |  |
| 10. When I feel unpleasant body sensations, I occupy myself with something else so I don’t have to feel them. |  | 0.74 | 0.04 | 19.97*** |  |
| **Not Worrying** |  |  |  |  |  |
| 11. When I feel physical pain, I become upset. |  | 0.66 | 0.05 | 13.85*** |  |
| 12. I start to worry that something is wrong if I feel any discomfort. |  | 0.62 | 0.04 | 17.01*** |  |
| 13. I can notice an unpleasant body sensation without worrying about it. |  | 0.68 | 0.04 | 16.60*** |  |
| 14. I can stay calm and not worry when I have feelings of discomfort or pain. |  | 0.76 | 0.04 | 22.01*** |  |
| 15. When I am in discomfort or pain, I can’t get it out of my mind. |  | 0.61 | 0.05 | 15.21*** |  |
| **Attention Regulation** |  |  |  |  |  |
| 16. I can pay attention to my breath without being distracted by things happening around me. |  | 0.61 | 0.05 | 15.98*** |  |
| 17. I can maintain awareness of my inner bodily sensations even when there is a lot going on around me. |  | 0.66 | 0.04 | 18.21*** |  |
| 18. When I am in conversation with someone, I can pay attention to my posture. |  | 0.56 | 0.04 | 14.66*** |  |
| 19. I can return awareness to my body if I am distracted. |  | 0.77 | 0.03 | 22.85*** |  |
| 20. I can refocus my attention from thinking to sensing my body. |  | 0.78 | 0.04 | 22.90*** |  |
| 21. I can maintain awareness of my whole body even when a part of me is in pain or discomfort |  | 0.63 | 0.04 | 16.37*** |  |
| 22. I am able to consciously focus on my body as a whole. |  | 0.63 | 0.04 | 17.12*** |  |
| **Emotional Regulation** |  |  |  |  |  |
| 23. I notice how my body changes when I am angry |  | 0.51 | 0.05 | 12.92*** |  |
| 24. When something is wrong in my life, I can feel it in my body. |  | 0.47 | 0.05 | 11.35*** |  |
| 25. I notice that my body feels different after a peaceful experience. |  | 0.72 | 0.05 | 18.10*** |  |
| 26. I notice that my breathing becomes free and easy when I feel comfortable. |  | 0.71 | 0.04 | 17.87*** |  |
| 27. I notice how my body changes when I feel happy / joyful. |  | 0.80 | 0.04 | 23.47*** |  |
| **Self-Regulation** |  |  |  |  |  |
| 28. When I feel overwhelmed, I can find a calm place inside. |  | 0.54 | 0.05 | 13.65*** |  |
| 29. When I bring awareness to my body, I feel a sense of calm. |  | 0.65 | 0.04 | 15.80*** |  |
| 30. I can use my breath to reduce tension. |  | 0.84 | 0.04 | 24.74*** |  |
| 31. When I am caught up in thoughts, I can calm my mind by focusing on my body/breathing. |  | 0.86 | 0.05 | 27.15*** |  |
| **Body Listening** |  |  |  |  |  |
| 32. I listen for information from my body about my emotional state. |  | 0.79 | 0.04 | 23.59*** |  |
| 33. When I am upset, I take time to explore how my body feels. |  | 0.78 | 0.04 | 23.55*** |  |
| 34. I listen to my body to inform me about what to do. |  | 0.74 | 0.04 | 19.98*** |  |
| **Trusting** |  |  |  |  |  |
| 35. I am at home in my body. |  | 0.87 | 0.04 | 24.14*** |  |
| 36. I feel my body is a safe place. |  | 0.92 | 0.04 | 27.82*** |  |
| 37. I trust my body sensations. |  | 0.67 | 0.04 | 16.69*** |  |
| *Note. p-value <0.05 = *, p-value<0.001 = **, p-value<0.001 = ****  *X^2^ = 1812.799, df = 601, p < 0.001; RMSEA = 0.60 [0.057]; SRMR = 0.067; CFI = 0.855; TLI = 0.839* | | | | |  |
